# Leukoencephalopathy following stereotactic radiosurgery for breast cancer brain metastases: a single-center analysis of 1,077 lesions

**DOI:** 10.1007/s00701-025-06478-8

**Published:** 2025-03-14

**Authors:** Salem M. Tos, Bardia Hajikarimloo, Georgios Mantziaris, Mariam Ishaque, Purushotham Ramanathan, David Schlesinger, Jason P. Sheehan

**Affiliations:** 1 Department of Neurosurgery, University of Virginia, Box 800212, Charlottesville, VA 22908 USA; 2 Department of Radiation Oncology, University of Virginia, Charlottesville, VA USA

**Keywords:** Breast cancer, Brain metastasis, Radiosurgery, Leukoencephalopathy

## Abstract

**Background:**

Leukoencephalopathy is the most prevalent and delayed adverse radiation effect (ARE) after intracranial radiotherapy (RT). Patients with leukoencephalopathy experience some levels of cognitive and neurological dysfunction. This investigation assessed the frequency and clinical outcomes of leukoencephalopathy following stereotactic radiosurgery (SRS) alone or SRS following whole-brain radiation therapy (WBRT) in breast cancer brain metastasis.

**Methods:**

We retrospectively evaluated the data of brain metastases from breast cancer individuals who underwent SRS between 2007 and 2022. MRI sequences were examined to assess white matter changes and tumor control.

**Results:**

Among 125 patients with 1,077 brain metastases, 58 (46.4%) patients received WBRT prior to SRS. By year 3, 23.4% of WBRT + SRS patients developed high-grade leukoencephalopathy (grades 2–3) compared to 5.7% in the SRS-only group (*p* < 0.001). In univariate analyses, significant predictors of high-grade leukoencephalopathy included prior WBRT (HR: 18.4, *p* = 0.005), cumulative integral dose > 3 J (HR: 4.17, *p* = 0.029), and the total number of lesions (HR: 1.22, *p* < 0.001). In multivariate analyses, prior WBRT (HR: 11.1, *p* = 0.022) and total lesions (HR: 1.14, *p* = 0.037) remained significant predictors.

**Conclusion:**

Our findings demonstrated that WBRT plus SRS is associated with higher leukoencephalopathy rates than SRS alone. This underscores the importance of carefully weighing the benefits and risks of different ionizing radiation approaches in the management of brain metastasis from breast cancer.

## Introduction

Breast cancer is one of the most frequently diagnosed malignancies, with an annual rate of occurrence of 2.26 million cases [2]. The brain is a prevalent metastasis location for breast cancer, and the incidence of metastatic lesions is documented to be 7% to 16% upon diagnosis [15]. Whole-brain radiation therapy (WBRT) has been used in the management of brain metastases since its introduction in the 1950s [15]. Several more advanced radiotherapy (RT) modalities have emerged due to the considerable neurocognitive impairments following WBRT, including hippocampal-avoidance WBRT, proton beam therapy, and intensity-modulated radiotherapy (IMRT) [2, 15, 18]. In recent decades, stereotactic radiosurgery (SRS) has been introduced and used extensively in brain metastasis [2]. SRS can deliver high doses of radiation to a focused region, enhancing radiological outcomes [2, 15, 18].

Leukoencephalopathy is the most frequent and threatening delayed adverse radiation effect (ARE) following intracranial RT [3]. Patients with leukoencephalopathy can experience some levels of dysfunction regarding memory, attention, concentration, learning, and executive function concurrent with imbalance and dementia [9, 15]. It is primarily correlated with two underlying mechanisms; vasculature obliterating sclerosis caused by endothelial injury, and subsequent ischemia and death of oligodendrocytes [4, 12, 14].

The goal of this retrospective single-center research was to assess the clinical and radiological outcomes of leukoencephalopathy following SRS in brain metastasis from breast metastases. This is the largest investigation to evaluate leukoencephalopathy in the setting of brain metastasis from breast cancer.

## Methods

### Study design

This retrospective cohort study included individuals with breast cancer brain metastasis who underwent SRS between 2007 and 2022 at our hospital. Patients who underwent multiple-fraction SRS, those without at least one follow-up MRI or clinical evaluation post-SRS, and those without high-quality MRI scans (including T1, T2, and FLAIR sequences) required for volumetric tumor analysis or accurate leukoencephalopathy grading were excluded. Ultimately, 125 patients with 1,077 brain metastases were enrolled in the study. The UVA hospital’s IRB authorized the investigation and waived informed consent due to its retrospective design. The study followed institutional ethical guidelines and the principles of the 1964 Helsinki Declaration and its amendments. Patient confidentiality was maintained throughout the data collection and analysis process. The study followed the STROBE guidelines [17].

### Data variables

Medical records were comprehensively reviewed to extract relevant data. Patient demographic and medical history parameters included “age at SRS”, “gender”, “Karnofsky Performance Status (KPS) score”, “recursive partitioning analysis (RPA)”, and histopathology data (“estrogen receptor (ER)”, “progesterone receptor (PR)”, “human epidermal growth factor receptor 2 (HER-2)”, “breast cancer gene (BRCA)” mutation status). This data was used to form a detailed profile of each patient and their tumor characteristics for further analysis. The presence of symptomatic brain metastasis was recorded at both the patient and lesion level. Additionally, detailed information about SRS parameters and intracranial disease burden, such as prescription dose of radiation, isodose line of the treatment plan, metastases treated per SRS session, and integral dose to the cranium (classified as ≤ 3 J and > 3 J), were collected from the radiosurgery treatment planning system (GammaPlan, Elekta Instrument, AB, Stockholm, Sweden) as previously described [15]. Patients were followed clinically and radiologically as part of institutional clinical protocols. Radiographic and clinical response outcomes included local tumor response (regression, stability, or progression depending on lesion and patient), new brain metastases, leptomeningeal spread, and survival. Secondary outcomes were subsequent treatment (repeat SRS, repeat WBRT, craniotomy & resection), KPS, and ECOG scores at the end of follow-up.

#### Clinical and radiological assessment after SRS

Magnetic resonance imaging (MRI) scans were conducted at baseline and every 3 months post-SRS and were reviewed by a neurosurgeon and neuroradiologist. Tumor response was assessed using volumetric analysis. Tumor response was assessed using volumetry on T1 post-contrast images, categorized as stable (± 10% of original volume), progression (> 10% increase), or regression (> 10% decrease). T1, T2, and FLAIR sequences were analyzed for white matter changes and lesion control. A simplified grading system was employed to categorize leukoencephalopathy, using a scale from 0 to 3. The grades were defined as follows: Grade 0 indicated normal appearance with no white matter changes, Grade 1 represented mild periventricular white matter changes, Grade 2 indicated moderate periventricular changes, and Grade 3 represented severe and/or diffuse white matter changes [15]. This system focused on diffuse periventricular alterations in FLAIR/T2 sequences, distinguishing them from localized tumor-associated edema [10, 16]. The progression of these grades was compared between patients receiving SRS alone and those undergoing both WBRT and SRS, with assessments conducted at baseline and years 1, 2, and 3 post-treatments.

#### Statistical analysis

All statistical computations were carried out using R Language in R Studio [13]. Data are summarized as median (IQR) for continuous variables and frequency (%) for categorical variables. Cox regression identified risk factors for high-grade (2–3) versus low-grade (0–1) leukoencephalopathy. Univariate analyses were conducted for variables including age at SRS, presence of symptomatic metastatic lesions, histology, receptor status, number of metastatic lesions, V12, total integral dose to the cranium, steroid use at the time of SRS, and pre-SRS therapies. Variables that were significant in the univariate analysis were then included in a multivariate analysis. The results were reported as hazard ratios (HR) with corresponding 95% confidence intervals (CI). The incidence of each leukoencephalopathy grade at each time point was calculated and visualized using an Alluvial plot, depicting annual changes for each year following the initial SRS. Fisher’s exact test and Pearson’s Chi-squared test were used to compare the SRS-only group and the WBRT + SRS group after dichotomizing leukoencephalopathy grades into low grade (0–1) and high grade (2–3). The statistical significance level was set at *p* ≤ 0.05.

## Results

### Patient and tumor characteristics

The median age at SRS was 54.0 years (IQR: 43.0–60.0) (Table 1). The vast majority of patients were female (98.4%, n = 123), with only two male patients (1.6%). Among the cases with known histology, 82 (84.5%) were invasive ductal carcinoma, and 15 (15.5%) were invasive lobular carcinoma. 57.6% of the cases were estrogen receptor-positive, 43.3% were PR-positive, and 37.6% were HER-2-positive. BRCA testing was available for a limited fraction of individuals (10 individuals), with one patient positive for BRCA-1 and nothing for BRCA-2. The most prevalent sites for metastases were the cerebellum (30.8%), frontal lobe (27.5%), and parietal lobe (13.1%). Notably, 73.0% of individuals had symptomatic brain metastases, accounting for 56.0% of all lesions.

**Table 1:** Patient and tumor characteristics

Characteristic	Patient (*N* = 125^*1*^)
Overall number of lesions	1,077
Age at SRS, Median (IQR)	54.0 (43.0, 60.0)
Gender, *n* (%)	
Female	123 (98.4%)
Male	2 (1.6%)
Histology, *n* (%)	
Invasive Ductal Carcinoma	82 (84.5%)
Invasive Lobular Carcinoma	15 (15.5%)
Unknown, patients (lesions)	28 (284)
Estrogen Receptor, *n* (%)	
Negative	42 (42.4%)
Positive	57 (57.6%)
Unknown, patients (lesions)	26 (178)
Progesterone Receptor, *n* (%)	
Negative	55 (56.7%)
Positive	42 (43.3%)
Unknown, patients (lesions)	28 (207)
HER-2, n (%)	
Negative	63 (62.4%)
Positive	38 (37.6%)
nknown, patients (lesions)	24 (159)
BRCA-1, *n* (%)	
Negative	4 (80.0%)
Positive	1 (20.0%)
Unknown, patients (lesions)	120 (1045)
BRCA-2, *n* (%)	
Negative	5 (100.0%)
Unknown, patients (lesions)	120 (1047)
Location, *n* (%)	
Cerebellum	327 (30.8%)
Frontal lobe	292 (27.5%)
Parietal lobe	139 (13.1%)
Occipital lobe	126 (11.9%)
Temporal lobe	86 (8.1%)
Brainstem	44 (4.1%)
Basal ganglia	19 (1.8%)
Thalamus	14 (1.3%)
Ventricular	6 (0.6%)
Hypothalamus	3 (0.3%)
Pituitary	3 (0.3%)
Corpus callosum	2 (0.2%)
Cavernous sinus	1 (0.1%)
Insula	1 (0.1%)
Unknown	14
Symptomatic brain metastasis, *n* (%)	
Per Patient	89 (73.0%)
Per Lesion	568 (56.0%)
Unknown, patients (lesions)	3 (63)
Recursive Partitioning Analysis, class, *n* (%)	
2	124 (99.2%)
3	1 (0.8%)
Pre-SRS resection, *n* (%)	
Per Patient	16 (12.8%)
Per Lesion	34 (3.2%)
Pre-SRS WBRT, *n* (%)	
Per Patient	58 (46.4%)
Per Lesion	646 (60.0%)
Concurrent Chemotherapy, *n* (%)	
Per Patient	72 (57.6%)
Per Lesion	647 (60.1%)
KPS score at SRS, *n* (%)	
60	1 (0.8%)
70	7 (5.6%)
80	30 (24.0%)
90	46 (36.8%)
100	41 (32.8%)
ECOG at SRS, *n* (%)	
0	41 (32.8%)
1	76 (60.8%)
2	8 (6.4%)
Number of mets per treatment, Median (IQR)	3.0 (1.0, 7.0)
Diameter (cm), Median (IQR)	6.0 (4.0, 10.1)
Volume of met (cm^3^), Median (IQR)	0.1 (0.0, 0.3)
Prescription dose (Gy), Median (IQR)	18.0 (17.0, 20.0)
Isodose line (%), Median (IQR)	75.0 (50.0, 95.0)
V12 (cc), Median (IQR)	0.7 (0.5, 1.5)
Integral dose (J), *n* (%)	
≤ 3	64 (51.2%)
> 3	61 (48.8%)
Leukoencephalopathy prior SRS, *n* (%)	39 (31.2%)
Leukoencephalopathy prior SRS grade, *n* (%)	
0	86 (68.8%)
1	36 (28.8%)
2	3 (2.4%)
Steroid at SRS, *n* (%)	59 (47.2%)
Last clinical follow up (months), Median (IQR)	25 (10–54)
Last Radiological follow up (months), Median (IQR)	27 (10–55)

### Treatment characteristics

The median prescription dose was 18.0 Gy (IQR: 17.0–20.0), with a median isodose line of 75.0% (IQR: 50.0–95.0). The median number of metastases treated per radiosurgical session was 3.0 (IQR: 1.0–7.0). Prior to SRS, 12.8% of patients had undergone surgical resection for at least one lesion, and 46.4% had received WBRT. Concurrent chemotherapy was administered in 57.6% of patients. At the time of SRS, 47.2% of individuals were on steroids.

### Radiological and clinical outcomes

Tumor regression occurred in 66 patients (51.6%) and 483 lesions (44.8%), while stability was seen in 33 patients (25.8%) and 388 lesions (36.0%) (Table 2). Progression was observed in 29 patients (22.7%) and 206 lesions (19.2%). New brain metastases developed in 99 patients (79.2%) during follow-up, and leptomeningeal dissemination occurred in 22 cases (17.6%). The overall mortality rate was 72.8%. Further treatments were required in some patients, including repeat SRS in 10 patients (8.0%), WBRT post SRS in 30 patients (24.0%), and resection in 5 patients (4.0%).

**Table 2:** Outcomes

Characteristic	Value, *n* (%)
Metastasis local response	
Progression	
Per patient	29 (22.7%)
Per lesion	206 (19.2%)
Regression	
Per patient	66 (51.6%)
Per lesion	483 (44.8%)
Stable	
Per patient	33 (25.8%)
Per lesion	388 (36.0%)
New brain metastasis lesion	99 (79.2%)
Leptomeningeal dissemination	22 (17.6%)
KPS at last follow up	
0	91 (72.8%)
50	2 (1.6%)
70	3 (2.4%)
80	10 (8.0%)
90	11 (8.8%)
100	5 (4.0%)
ECOG at last follow up	
0	5 (4.0%)
1	21 (16.4%)
2	3 (2.4%)
3	2 (1.6%)
4	0 (0.0%)
5	94 (75.2%)
Status at last follow up	
Alive	10 (8.0%)
Dead	91 (72.8%)
Lost to follow-up (Unknown)	24 (19.2%)
Cause of death	
Brain metastases	7 (7.7%)
Others	23 (25.3%)
Unknown	61 (67%)
Further treatment	
SRS	10 (8.0%)
WBRT	30 (24.0%)
Resection	5 (4.0%)

#### Leukoencephalopathy progression: SRS vs. SRS following WBRT

**Fig. 1:**
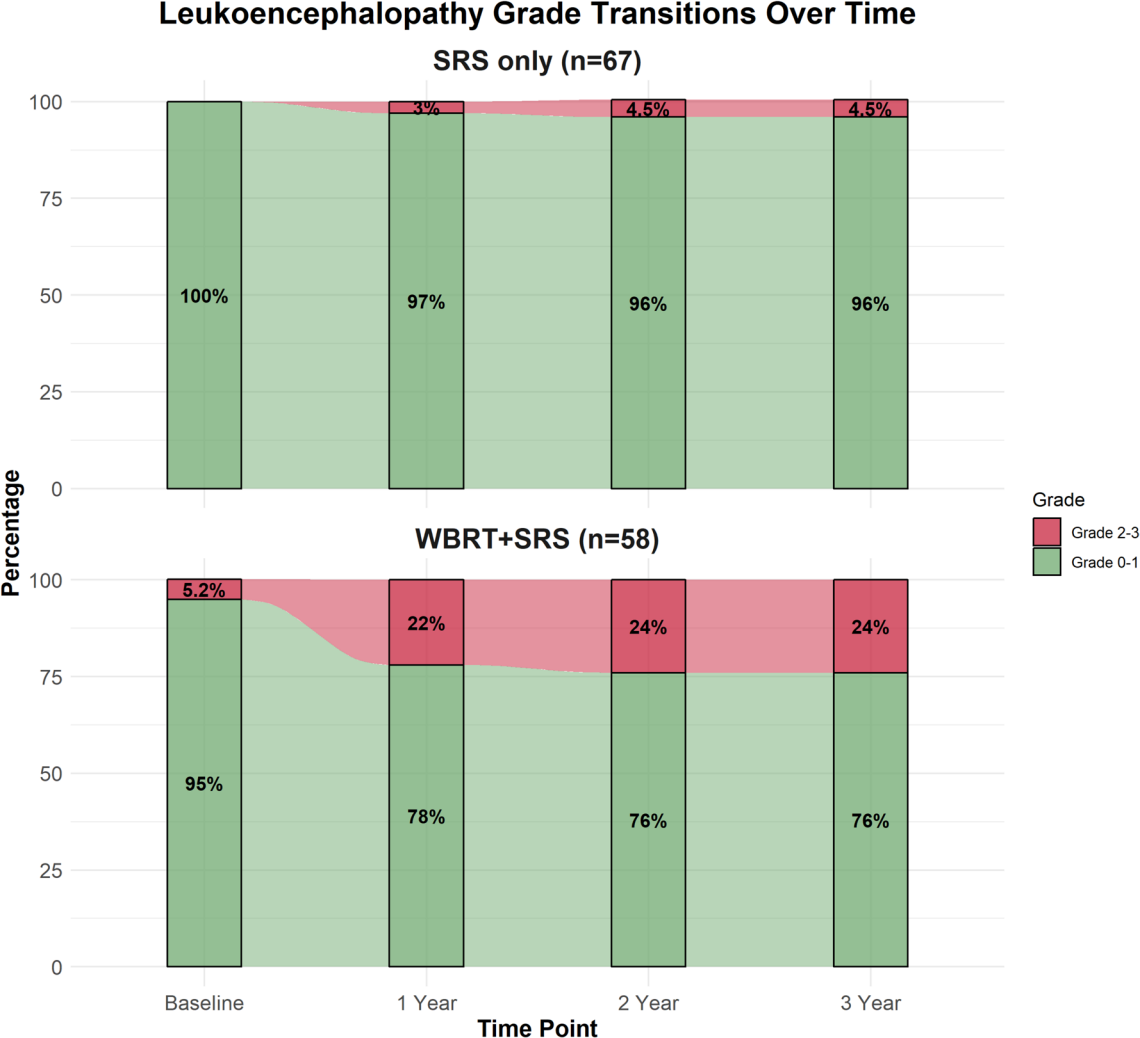
The transitions of leukoencephalopathy grades over time for patients treated with SRS only vs WBRT followed by SRS

The transitions of leukoencephalopathy grades over time for patients treated with SRS only (*n* = 67) and WBRT + SRS (*n* = 58) are shown in Fig. 1. Grades were dichotomized into low grade (0–1) and high grade (2–3). At baseline, the proportion of patients with low-grade leukoencephalopathy was 100% in the SRS-only and 95% in the WBRT + SRS groups. After dichotomization, this baseline variation was not significant (*p* = 0.10). At 1 year, the proportion of patients with low-grade leukoencephalopathy decreased to 97% in the SRS-only group and 78% in the WBRT + SRS group, with a significant difference (*p* < 0.001). By 2 and 3 years, the proportion of patients with low-grade leukoencephalopathy remained at 96% in the SRS-only and declined to 76% in the WBRT + SRS groups. The variation across the group was significant at both time points (*p* = 0.001).

The predictors of high-grade leukoencephalopathy (grades 2–3) compared to low-grade (grades 0–1) are summarized in Table 3. On univariable analysis, the total number of metastatic lesions (HR: 1.22, 95% CI: 1.10–1.35, *p* < 0.001), integral dose > 3 J (HR: 4.17, 95% CI: 1.16–15.0, *p* = 0.029), and prior WBRT before SRS (HR: 18.4, 95% CI: 2.40–141, *p* = 0.005) were significant. On multivariable analysis, the total number of metastatic lesions (HR: 1.14, 95% CI: 1.01–1.29, *p* = 0.037) and prior WBRT (HR: 11.1, 95% CI: 1.41–87.8, *p* = 0.022) remained significant. Other factors, including age, histology, hormone receptor status, HER2 status, and pre-SRS treatments, were not significant in either analysis.

**Table 3:** Cox regression analysis comparing low-grade (grades 0–1) vs. high-grade (grades 2–3) leukoencephalopathy

	Univariable				Multivariable		
Characteristic	N	HR^*1*^	95% CI^*1*^	*p*-value	HR^*1*^	95% CI^*1*^	*p*-value
Age at SRS	125	1.02	0.97, 1.07	0.44			
Symptomatic metastatic lesions	122						
No		—	—				
Yes		2.45	0.55, 11.0	0.24			
Histology (IDC/ILC)	97						
Invasive ductal carcinoma		—	—				
Invasive lobular carcinoma		0.60	0.08, 4.71	0.62			
Estrogen receptor	99						
Negative		—	—				
Positive		1.26	0.37, 4.31	0.71			
Progesterone receptor	97						
Negative		—	—				
Positive		1.58	0.48, 5.19	0.45			
HER2	101						
Negative		—	—				
Positive		2.12	0.67, 6.70	0.20			
Total metastatic lesions (reference: 1 lesion)	125	1.22	1.10, 1.35	** < 0.001**	1.14	1.01, 1.29	**0.037**
V12 (cc)	110	0.93	0.83, 1.05	0.23			
Integral dose (J)	125						
≤ 3		—	—		—	—	
> 3		4.17	1.16, 15.0	**0.029**	1.74	0.44, 6.80	0.43
Steroids at SRS	125						
No		—	—				
Yes		1.31	0.46, 3.79	0.61			
Pre-GKS resection	125						
No		—	—				
Yes		0.38	0.05, 2.91	0.35			
Pre-GKS WBRT	125						
No		—	—		—	—	
Yes		18.4	2.40, 141	**0.005**	11.1	1.41, 87.8	**0.022**
Pre-GKS chemotherapy	125						
No		—	—				
Yes		1.11	0.37, 3.32	0.85			

^*1*^*HR *Hazard Ratio, *CI *Confidence Interval

Bold values indicate statistical significance

Analysis compares low-grade leukoencephalopathy (grades 0–1: none to minimal) with high-grade leukoencephalopathy (grades 2–3: moderate to severe)

## Discussion

### Incidence and severity of leukoencephalopathy

Our study on leukoencephalopathy in individuals with brain metastasis from breast cancer enhances the growing evidence regarding the incidence and severity of this condition following radiation therapy. We observed significant differences in leukoencephalopathy rates and severity between individuals who received SRS alone and those receiving WBRT plus SRS, consistent with previous studies across various cancer types. Following ionizing radiation, a major cause of brain injury is likely the induction of neural inflammation and activation of microglia. Cellular mediators of radiation-induced neurotoxic inflammation include interleukin-8 (IL-8) and tumor necrosis factor-alpha (TNF-α). Such radiation-induced inflammation can occur during or in a delayed fashion, resulting in neurocognitive side effects and chronic white matter loss in individuals. Radiologically, these phenomena can present as inflammation following ionizing irradiation and varying degrees of leukoencephalopathy months to years subsequent radiation delivery [11].

The incidence of leukoencephalopathy in our cohort showed a clear distinction between treatment groups. At baseline, the proportion of patients with no or low-grade leukoencephalopathy was 100% in the SRS-only and 95% in the WBRT + SRS group (*p* = 0.10). At 1 year, the proportion of patients with no or low-grade leukoencephalopathy decreased to 97% in the SRS-only and 78% in the WBRT + SRS group, with a significant difference (*p* < 0.001). By 2 and 3 years, the proportion of patients with no or low-grade leukoencephalopathy remained at 96% in the SRS-only and declined to 76% in the WBRT + SRS group. The difference between the groups was statistically significant at both time points (*p* = 0.001). These findings align with previous research, such as Monaco et al. study on lung carcinoma individuals, which reported significantly higher rates of leukoencephalopathy in individuals who received WBRT plus SRS (97.3%) compared to SRS alone (3.2%) [10]. Our results also show similarities to the study by Trifiletti et al., which reported leukoencephalopathy rates of 29%, 38%, and 53% at years 1, 2, and 3, respectively, in patients treated with multiple SRS sessions [15]. In another study by Liu et al., they reported that in a cohort of 993 patients with brain metastases, encompassing 291 WBRT + SRS and 702 SRS alone, the overall incidence rate of leukoencephalopathy was 20.1% [8]. They also showed that the leukoencephalopathy incidence rates were significantly higher across patients who revied WBRT plus SRS than SRS alone at 1-year (19% vs. 2.6%), 2-year (45.9% vs. 5.3%), 3-year (67.5% vs. 7.8%), and 5-year (85% vs. 14.7%) follow-ups (*P* < 0.001) [8]. Cohen-Inbar et al., in a study of 92 brain metastasis patients, showed that the incidence of leukoencephalopathy was higher in the WBRT plus SRS than in SRS alone at 1-year (*P* = 0.0002) and 2-year (*P* = 0.0345) follow-ups [5]. Similar to our study, Choi et al. exhibited that in 63 melanoma cases with brain metastasis, the incidence of grade 2 or 3 leukoencephalopathy was significantly greater in the WBRT plus SRS than in SRS (71.4% vs. 12.2%, *P* < 0.0001) [1]. Our observation of increasing leukoencephalopathy prevalence over time, with 18.8% of SRS following WBRT patients progressing to grade 2 or 3 leukoencephalopathy by year 3 (compared to only 4.5% in the SRS group), underscores the need for extended follow-up and consideration of long-term quality of life in treatment decisions.

### Prognostic factors of leukoencephalopathy

Analysis of risk factors for leukoencephalopathy development revealed important insights. In the univariable analysis, the number of metastatic tumors was significantly correlated with an escalated risk of high-grade leukoencephalopathy (HR: 1.22, 95% CI: 1.10–1.35, *p* < 0.001). Additionally, a cumulative integral dose exceeding 3 J indicated poorer outcomes (HR: 4.17, 95% CI: 1.16–15.0, *p* = 0.029). However, the most striking finding was the impact of pre-SRS WBRT, which emerged as a strong predictor of high-grade leukoencephalopathy in both univariable (HR: 18.4, 95% CI: 2.40–141, *p* = 0.005) and multivariable analyses (HR: 1.14, 95% CI: 1.01–1.29, *p* = 0.037). Trifiletti et al. demonstrated that the total number of treated lesions (HR: 1.084, *P* = 0.003), the cumulative integral dose of SRS to the cranium (HR: 1.273, *P* = 0.036), and WBRT prior to SRS (HR: 2.564, *P*= 0.019) were correlated with an increase in the likelihood of leukoencephalopathy following irradiation [15]. Similarly, Cohen-Inbar et al. demonstrated that an integral dose to the skull greater than 3 J (HR: 3.28, = 0.012) and pre-SRS WBRT (HR: 2.825, *P* = 0.042) were accompanied by a greater leukoencephalopathy risk, while the total number of treated lesions were not significantly accompanied by the greater risk of leukoencephalopathy (HR: 1.030, *P*= 0.663) [5]. Liu et al. exhibited that ages older than 77 years (HR: 2.971, *P* < 0.001), lesions larger than 28 cm^3^ (HR: 6.271, *P* < 0.001), and a combination of WBRT and SRS (HR: 11.97, *P *< 0.001) were correlated with a greater likelihood of leukoencephalopathy occurrence [8]. In contrast, their findings suggested that prior chemotherapy (HR: 1.397, *P* = 0.166) and the multiplicity of treated lesions (HR: 1.172, *P *= 0.303) were not associated with statistically greater leukoencephalopathy development [8]. Notably, several factors that have been implicated in previous studies showed no significant association with leukoencephalopathy outcomes in our cohort. These included age, symptomatic metastatic lesions presentation, histology, receptor status, 12 Gy volume (V12) for SRS, steroid use at SRS, pre-SRS resection, and pre-SRS chemotherapy. For example, Ebi et al., who found older age to be a significant risk factor, and Conill et al., who identified pre-existing leukoaraiosis as a prognostic factor for further leukoencephalopathy development. [6, 7] This discrepancy might be due to differences in patient populations or treatment protocols and warrants further investigation in future studies.

## Limitations

Our study has several limitations. The data were collected from a single center, which may restrict the generalizability of the results due to differences in treatment protocols and follow-ups across various institutions. The study’s retrospective nature is another limitation that makes it susceptible to the introduction of selection bias that may affect the reliability of outcomes. Lack of assessment of the potential influence of the concurrent chemotherapeutic agents that vary in regimen, type, and dose on leukoencephalopathy development or alterations is another limitation of our study. An important limitation of our study is the differences in follow-up duration, which directly impact the incidence and long-term clinical and radiological outcomes of leukoencephalopathy. Also, while we did assess radiological findings of leukoencephalopathy on MRI, we did not correlate these findings with specific neurocognitive or other neurological findings in this retrospective study. Another limitation is that we did not systematically gather or analyze data on whether individuals received hippocampal-sparing WBRT or memantine, leaving a critical gap in understanding how these modalities might have affected the findings.

## Conclusion

In conclusion, our study contributes valuable data on leukoencephalopathy in breast cancer brain metastases, supporting the broader literature on radiation-induced white matter changes. The significantly higher rates and severity of leukoencephalopathy in patients receiving WBRT plus SRS, compared to SRS alone, underscore the importance of carefully weighing the benefits and risks of different radiation approaches in the management of breast cancer brain metastases. Our results strongly suggest that avoiding WBRT when possible, may significantly reduce the risk of high-grade leukoencephalopathy in breast cancer patients with brain metastases. However, this must be balanced against the potential benefits of WBRT in certain clinical scenarios. Future research should focus on identifying subgroups of patients who might benefit most from WBRT while minimizing the risk of severe leukoencephalopathy.

## Data Availability

No datasets were generated or analysed during the current study.
